# Day and night heart rate variability using 24-h ECG recordings: a systematic review with meta-analysis using a gender lens

**DOI:** 10.1007/s10286-023-00969-3

**Published:** 2023-08-04

**Authors:** Beatrice De Maria, Monica Parati, Laura Adelaide Dalla Vecchia, Maria Teresa La Rovere

**Affiliations:** 1 Istituti Clinici Scientifici Maugeri IRCCS, Via Camaldoli 64, 20138 Milano, Italy; 2 Department of Electronics, Information and Bioengineering, Politecnico di Milano, Milan, Italy; 3 Istituti Clinici Scientifici Maugeri IRCCS, Montescano, Italy

**Keywords:** Heart rate variability, Sex difference, Gender difference, Night

## Abstract

**Purpose:**

Increasing evidence demonstrates that gender-related factors, and not only biological sex, are relevant in the physiological and pathophysiological mechanisms of the cardiovascular system, including the cardiac autonomic regulation. Sex and gender may also affect daytime and night-time cardiac autonomic control. This meta-analysis aimed to provide a comparison between healthy women and men on heart rate variability using 24-h ECG recordings pointing out sex- and gender-related factors.

**Methods:**

A systematic search was conducted to include studies focusing on both sex and gender differences related to heart rate variability indices in the time and frequency domains. Descriptive data were extracted by two independent reviewers. For each index, standardized mean differences with 95% confidence intervals were computed and a pooled estimate using a fixed- or random-effects model was applied.

**Results:**

Twenty-seven studies were included in the meta-analysis. The results showed that only seven studies reported some information about gender-related factors. Concerning sex-related differences, women had a shorter mean RR interval and lower variability of the time domain indices than men.

Sex-related differences concerning frequency domain indices were more evident during night-time compared to daytime.

**Conclusion:**

The characterization of gender-related factors in the study of heart rate variability using 24-h ECG recordings is still sporadic and underexplored. The meta-analysis results could not conclusively support a significant increase of high frequency power in women, although women showed a reduced total power and low frequency to high frequency ratio. There is a strong need for considering heart rate variability in relation to gender-related variables.

**Supplementary Information:**

The online version contains supplementary material available at 10.1007/s10286-023-00969-3.

## Introduction

It is well known that biological sex-related differences are relevant in a number of physiological and pathophysiological conditions, including cardiovascular ones [1–3]. However, psycho-socio-demographic factors causing different familial and societal engagement, usually referred to as gender-related factors, could also have an impact. While it is recognized that sex hormones influence the cardiovascular risk, it is conceivable that gender-related factors may also have an impact on the cardiovascular risk [4].

Nowadays, systematic sex-disaggregated data collection (i.e. data reported separately for male and female individuals) and their interpretation are still scarce, standardized methods for quantifying gender-related factors are lacking, and understanding of the relationships between sex and gender is largely incomplete [5], leading to a lack of specific and individualized treatments.

Over the past 30 years, the study of cardiac nervous control through the analysis of heart rate variability (HRV) has made it possible to investigate the etiopathogenesis of numerous cardiovascular and non-cardiovascular conditions and identify several prognostic indices; however, once again, sex-disaggregated data are scattered and the possible influence of gender is even more incomplete. HRV can be assessed over short periods of around 5 min under laboratory and controlled conditions, usually referred to as short-term HRV, and over longer recordings of about 24 h. In 2016, a meta-analysis [6], which mainly focused on short-term recordings and also included some analysis derived from 24-h recordings, showed that the autonomic control of the heart features some differences in female and male individuals, the former being characterized by relative dominance of vagal activity, despite greater mean heart rate (HR), and the latter by relative sympathetic dominance, despite lower HR. Moreover, sex-related differences are attenuated by ageing [7]. In addition, some studies evidenced the dependence of sex-related differences on the considered period, i.e. daytime or night-time [8]. A recent prospective comparative study of healthy subjects [9] has demonstrated that the autonomic cardiac control adjusts differently in response to the same physiological perturbations in women compared to men during daytime and night-time.

In order to consider both day- and night-time analyses, in addition to the fact that HRV derived from short or 24-h recordings is not directly comparable because of the different duration of the recordings [10], the present systematic review focuses only on 24-h recordings. The general aim is to provide an updated comparison between healthy women and men investigated by means of HRV analysis derived from 24-h electrocardiogram (ECG) recordings pointing out sex-related and gender-related influencing variables, day and night differences, and age-related influences.

In detail, the specific aims are (1) to compare the time and frequency domain HRV indices in male and female individuals; (2) to compare time and frequency domain HRV indices in men and women separately during daytime and night-time; (3) to assess whether differences between men and women are influenced by age; (4) to point out information about gender-related factors reported in the available studies that assess sex differences in HRV.

## Methods

### Protocol

The review was conducted in accordance with PRISMA (Preferred Reporting Items for Systematic Reviews and Meta-analysis) statements. The review protocol was not recorded in an online register.

### Database and search criteria

Three electronic databases (Pubmed, Embase, Scopus) were searched until 31 January 2023. Methodology search filters by study design, language or publication date were not applied to possibly minimize retrieval biases. The following keywords and related MeSH/Entree terms used in a previous systematic review on this topic [6] were appropriately combined using Boolean operators to create the search strategy for each database: sex, gender, gender identity, women, men, female, male, heart rate variability, heart period variability, HRV. Reference lists of the included studies were finally screened to identify any further related articles to be included.

### Eligibility criteria

The inclusion criteria for this systematic review were:

1. Articles assessing individuals with an age higher than 18 years.
2. Subjects without pathologies and medication.
3. ECG recordings ≥ 24 h.
4. Outcome measures of HRV in time and frequency domain.
5. Outcome measures separately reported from male and female populations.
6. Sufficient quantitative data to perform meta-analysis technique between male and female groups (i.e. number of samples and descriptive statistics or individual data).

The exclusion criteria were:

1. Data related to athletes.
2. Reviews, meta-analyses, abstracts and comments.

### Study selection and data extraction

All the retrieved records were merged and screened to remove duplicates. After duplicates were removed, the title and abstracts of all identified articles were independently evaluated by two reviewers (MP, BDM). Full texts of all potentially relevant articles were retrieved and their eligibility for this review was examined. The number of articles meeting the eligibility criteria, the number of excluded studies and the reasons for exclusion were recorded. Relevant data from eligible articles were extracted in a structured data extraction form by a reviewer (MP) and their accuracy was checked by a second reviewer (BDM). The relevant data mainly include anthropometric characteristics of participants, gender-related factors (i.e. any variable better describing the socio-economic status, behavioural and psychological conditions), experimental study design and outcome data. Any disagreement between the two reviewers during the retrieval process was resolved by discussion until a consensus was reached.

### Data analysis

The HRV outcome indices included in this systematic review are listed and briefly explained in Table 1. In this review, only the internationally recognized indices for which the interpretation is univocal were considered. The standard deviation of the RR interval (SDNN) and the total power (TP) of the RR series were taken as overall indices of the amplitude of HRV oscillations [10]. The root mean square of successive differences between adjacent RR interval (RMSSD), the absolute and normalized power in the high-frequency band (HF, 0.15–0.40 Hz), labelled HF and HFnu, respectively, were taken as indices of the cardiac vagal modulation [10, 11]. The ratio of the RR power in the low-frequency band (LF, 0.04–0.15 Hz) to the HF power was taken as an index of the cardiac sympatho-vagal balance [10, 11]. For each HRV outcome, the measurements in each individual study were collected and combined whenever possible through meta-analysis techniques [12]. The HRV measures were treated as continuous data and therefore the mean, standard deviation and number of analysed participants in the two groups (male vs female) were recorded. Missing means and standard deviations in the individual studies were estimated by means of imputation from available data [12, 13]. If the measurements of an outcome were presented in the original studies as subgroups (e.g. data shown subdivided in age bins), their values were extracted and combined using the weighted means and the pooled standard deviations [12, 13].

**Table 1:** Outcome measures

Time domain analysis	
RR (ms)	Mean time interval between two consecutive R waves in milliseconds
SDNN (ms)	Standard deviation of RR intervals in milliseconds
RMSSD (ms)	Root mean square of successive differences between adjacent RR intervals in milliseconds
Frequency domain analysis	
TP (ms^2^)	Total power of RR series expressed in milliseconds squared
HF (ms^2^)	Power of RR series in the high frequency band (0.15–0.40 Hz) in milliseconds squared
HFnu (nu)	Power of RR series in the high frequency band (0.15–0.40 Hz) in normalized units
LF/HF	Ratio between the low- (0.04–0.15 Hz) and high-frequency components of RR series

Regarding the first objective, the HRV indices derived from 24-h ECG recordings were considered in the analyses. If an article examined 24-h recordings and separately reported the measurement during daytime and night-time, their values were combined using weighted mean and pooled standard deviation [12].

As to the second study aim, the indices computed during daytime and night-time were separately examined.

The effect measures were estimated through the standardized mean difference (SMD) based on Hedges’* g*, along with the 95% confidence interval (CI) [12, 14]. Heterogeneity among studies was evaluated using the *I*^2^ statistics and the *χ*^2^ test [12, 15]. We calculated the overall effects using a random-effect model in case of high heterogeneity (> 50%) or a fixed-effect model otherwise [16]. A positive effect size conventionally means that the male population showed a higher value in the outcome measure compared to the female population.

As it is known that HRV decreases with ageing [17, 18], mixed-effect meta-regression analyses were used to analyse the influence of the mean subject age on the sex-related effect. In order to have sufficient power, meta-regression was only performed for HRV indices reported in at least ten studies [12].

The meta-analyses were performed using Review Manager 5.4 software [19]. The meta-regression analyses were computed using R 4.0 software. The null hypothesis of no statistical difference was rejected if the *p* value was less than 0.05.

## Results

### Search results and study characteristics

**Fig. 1:**
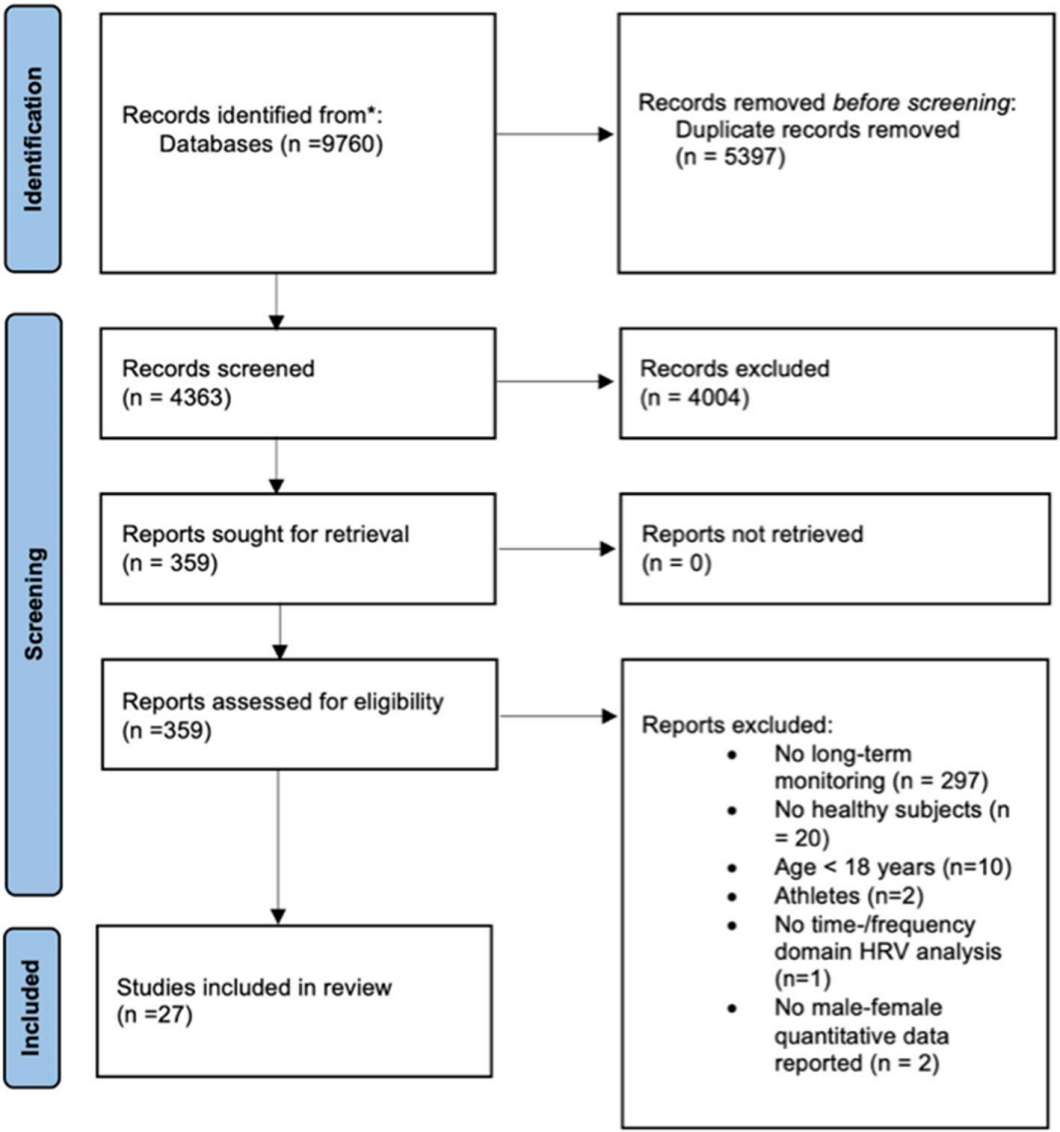
Flowchart of the study selection process

A total of 9760 articles were originally found in the literature. After duplicate and eligibility screening, 27 studies from 29 articles were included in this systematic review. The flow diagram of the study selection process is shown in Fig. 1.

The main characteristics of the included studies are shown in Table 2. Overall, they were published between 1991 and 2022. A total of 16 (59%) studies were conducted in Europe, 9 (33%) in America, 1 (4%) in Asia and 1 (4%) in Africa.

**Table 2:** Main characteristics of the included studies

Authors	Country	M,* n*	F,* n*	Age M, years	Age F, years	BMI M, kg/m^2^	BMI F, kg/m^2^	Sample type	Psychosocial variables	Length of recording	DAY vs NIGHT	System	SR, Hz	Time domain indices	Frequency domain indices
Beckers et al. [34]	Belgium	141	135	NR (18–71)	NR (18–71)	25 ± 3	23 ± 4	Healthy subjects	No	24 h	Yes	ELA Medical Holter	200	RR, SDNN, RMSSD	TP, HF, HFnu, LF/HF (FFT—iterated analysis, windows of 128 s, 50% overlap)
Bonnemeier et al. [46, 47]	Germany	85	81	43 ± 3 (20–70)	42 ± 3 (20–70)	23 ± 1	22 ± 1	Healthy healthcare professionals and their family members; healthy former patients with atypical chest pain	No	24 h	No	Tracker II, Reynolds, Hertford, UK	NR	RR, SDNN, RMSSD	NR
Bigger et al. [48]	USA	202	72	57 ± 8 (40–69)		NR	NR	Healthy employees and persons who responded to newspaper advertisement	No	24 h	No	Marquette 8000	NR	RR	NR
De Maria et al. [9, 49]	Italy	50	50	35 ± 5 (28–48)	37 ± 7 (25–49)	25 ± 3	23 ± 3	Healthy healthcare professionals	Yes	24 h	Yes	eMotion Faros 360°, Mega Electronics, Finland	500	RR	TP, HF (AR—iterated analysis, windows of 250 RRs, overlap of 200 RRs)
Extramiana et al. [50]	France	30	30	34 ± 9 (20–50)		NR	NR	Healthy subjects	No	24 h	Yes	Del Mar 459 Recorder, Del Mar Avionics, Irvine, California	NR	RR, SDNN, RMSSD	NR
Genovesi et al. [51]	Italy	20	20	29 ± 4 (NR)	27 ± 4 (NR)	23 ± 2	21 ± 2	Healthy sedentary subjects	No	24 h	Yes	ELA Medical, Le Plessis Robinson, France	200	RR, SDNN	NR
Hulsegge et al. [20]	Denmark	292	251	46 ± 10 (18–68)		27 ± 5		Healthy blue-collar workers	Yes	96 h	No	Actiheart monitor	NR	RR, SDNN RMSSD	TP, HF, LF/HF (NR—iterated analysis, windows of 300 s, no overlap)
Jensen-Urstad et al. [52]	Sweden	49	53	NR (20–69 years)		25 ± 3	23 ± 3	Healthy healthcare professionals, students and blood donors	No	24 h	No	Reynolds Sherpa, Reynolds Medical, Hertford, England	NR	RR, SDNN, RMSSD	TP, HF, LF/HF (AR—iterated analysis, windows of 300 s, no data about overlap)
Jensen-Urstad et al. [21]	Sweden	63	70	35 ± 0 (NR)		25 ± 2	23 ± 4	Healthy subjects	Yes	24 h	Yes	Reynolds Sherpa, Reynolds Medical, Hertford, England	NR	RR	TP, HF, LF/HF (AR—iterated analysis, windows of 300 s, no data about overlap)
Kashiwagi et al. [53]	Japan	15	17	49 ± 14 (NR)	50 ± 11 (NR)	NR	NR	Healthy subjects	No	48 h	Yes	NR	NR	NR	HFnu (NR—iterated analysis, windows of 512 RRs every 30 min)
Krauss et al. [54]	Germany	63	76	41 ± NR (20–77)	42 ± NR (20–77)	NR	NR	Healthy subjects	No	24 h	No	Tracker II, Delmar Reynolds, Hertford, UK	NR	RR, SDNN, RMSSD	NR
Lampert et al. [22]	USA	87	70	30 ± 1 (18–50)	29 ± 1 (18–50)	28 ± 1	28 ± 1	Healthy community dwelling subjects	Yes	24 h	No	GE Seer and Seer Lite	125	SDNN	NR
Li et al. [55]	USA	35	60	55 ± 8 (NR)	58 ± 8 (NR)	27 ± 3	27 ± 6	Healthy community dwelling subjects	No	24 h	No	Mortara Instrument, Inc., WI, USA	1000	RR, SDNN, RMSSD	LF/HF (FFT—iterated analysis, windows of 300 s, no overlap)
Lerma et al. [56]	Mexico	20	30	29 ± NR (21–36)	27 ± NR (21–36)	23 ± NR (23–32)	23 ± NR (22–27)	Healthy subjects	No	24 h	No	Burdik-Spacelabs, 92,513, Dreefield	200	RR, SDNN, RMSSD	TP, HF, HFnu, LF/HF (FFT—NR)
Marin-Farrona et al. [23]	Spain	47	66	39 ± 6	39 ± 6	25 ± 2	24 ± 2	Healthy office workers	Yes	48 h	No	Firstbeat Bodyguard device, Firstbeat Technologies Ltd., Jyväskylä, Finland	NR	RMSSD	NR
Molnar et al. [57]	USA	11	10	57 ± 12 (36–76)	57 ± 15 (36–76)	NR	NR	Healthy subjects	No	24 h	No	Marquette 8000 T Laser Holter	NR	RR	NR
Pitzalis et al. [58]	Italy	44	45	26 ± 6 (NR)	27 ± 4 (NR)	24 ± 2	21 ± 3	Normotensive subjects with and without family history of hypertension	No	24 h	No	Del Mar Avionics Model 445A, Irvine, California, USA	NR	RR, SDNN RMSSD	NR
Ramaekers et al. [30]	Belgium	141	135	41 ± 14 (18–71)	43 ± 15 (18–71)	25 ± 3	23 ± 4	Healthy subjects	No	24 h	No	NR	200	RR, SDNN, RMSSD	TP, HF, HFnu, LF/HF (FFT—iterated analysis, windows of 256 s, overlap of 50%)
Strauss et al. [24]	South Africa	284	396	25 ± 3 (NR)	25 ± 3 (NR)	25 ± 5	26 ± 6	Black and white healthy subjects	Yes	24 h	No	Card(X)plore, Meditech, Budapest, Hungary	NR	RR, SDNN	HFnu, LF/HF (NR—NR)
Sammito et al. [59]	Germany	319	376	40 ± 10 (NR)	40 ± 11 (NR)	27 ± 4	25 ± 4	Healthy office employees, healthcare professionals, students and military	No	24 h	No	Schiller MT-101, Firma Schiller AG, Baar, Switzerland	1000	SDNN, RMSSD	HFnu, LF/HF (FFT—iterated analysis, windows of 256 s, overlap of 50%)
Sztajzel et al. [60]	Switzerland	14	18	29 ± 3 (NR)		NR	NR	Healthy medical students, residents and technicians	No	24 h + 24 h	Yes	NR	NR	RR, SDNN, RMSSD	TP, HF, HFnu, LF/HF (FFT—NR)
Sosnowski et al. [61]	Poland	173	37	50 ± 6 (NR)		NR	NR	Healthy subjects	No	24 h	No	MEdilog Excel 2 System, Oxford Instruments, Abingdon, UK	NR	RR, SDNN	NR
Stein et al. [62]	USA	30	30	50 ± 4 (NR)		NR	NR	Healthy subjects	No	24 h	Yes	Marquette series 8500, Milwaukee, Wisconsin	NR	RR, SDNN, RMSSD	LF/HF (FFT—iterated analysis. Windows of 300 s, no overlap)
Stein et al. [25]	USA	18	18	37 ± 9 (23–59)		NR	NR	Healthy subjects	Yes	24 h	Yes	ACS Holter, Ontario, CA	NR	RR, SDNN, RMSSD	LF/HF (FFT—iterated analysis. Windows of 300 s, no overlap)
Sevre et al. [63]	Norway and Netherlands	19	15	54 ± 3 (NR)	51 ± 1 (NR)	26 ± 1	27 ± 1	Healthy subjects	No	24 h	No	Marquette series 8500	NR	RR	NR
Umetani et al. [64]	USA	65	81	NR (30–69)		NR	NR	Healthy subjects	No	24 h	No	Cardionostics Dura-Lite recorder (Cardionostics), Epicardia 4000 53A-01	NR	RR, SDNN, RMSSD	NR
Van Hoogenhuyze et al. [65]	USA	19	14	33 ± 7 (NR)	34 ± 8 (NR)	NR	NR	Healthy subjects	No	24 h	No	Marquette 8500	NR	RR, SDNN	NR

Data are reported as mean ± standard deviation. As to age, when declared, the age range is reported in brackets

*M* male, *F* female, *BMI* body mass index, *DAY* daytime, *NIGHT* night-time, *SR* sampling rate, *RR* RR interval, *SDNN* standard deviation of the RR intervals, *RMMSD* square root of the mean squared differences of successive RR intervals, *TP* total power, *HF* power of the RR series in the high frequency band (0.04–0.15 Hz), *HFnu* normalized HF, *LF/HF* LF to HF ratio, *FFT* fast Fourier transform, *AR* autoregressive model, *NR* not reported

The sample size of the studies varied between 21 and 695 enrolled subjects. The total number of individuals considering all included studies was 4592. The mean male-to-female ratio was 1.2 with a standard deviation of 0.8; the maximum ratio was 4.7 while the minimum was 0.6.

Just 15 studies (56%) reported the mean age separately for the male and female populations. The lower mean age of enrolled subjects was 25 ± 3 years, and the highest was 57 ± 8 years.

All included subjects were healthy. Some studies included healthcare professionals and hospital staff (15%, *n* = 4) or office employees (15%, *n* = 4). Only 7 studies (26%) reported some additional psychosocial variables of the studied population. These variables included occupation and lifestyle habits [9, 20], risk factor assessment [21], stress evaluation [9, 22, 23], work ability [23], socio-economic status [24], pain tolerance [25] and psychological measures related to functional health status, depression, anger and anxiety [25].

Most studies (85%, *n* = 23) were conducted using a 24-h ECG recording, and only 4 (15%) used multiday recordings. The used recording system was almost always described, while the sampling rate was reported only in 8 studies (30%). The results of the time domain analysis were reported in all but one study. The results of the frequency domain analysis were reported in 15 studies (56%).

As to the frequency domain analysis, details about the performed analysis are reported in the last column of Table 2. Eight studies estimated the power spectrum by means of fast Fourier transform, three applied an autoregressive model and three studies did not report this information. The majority of the studies performed an iterated analysis over short time windows ranging from 128 s to 8 min, while three studies did not report any information. The overlap ranged from no overlap to 80% overlap.

Data reported separately for daytime and night-time were available only in 9 (33%) studies.

### Effect sizes during 24-h recordings

**Fig. 2:**
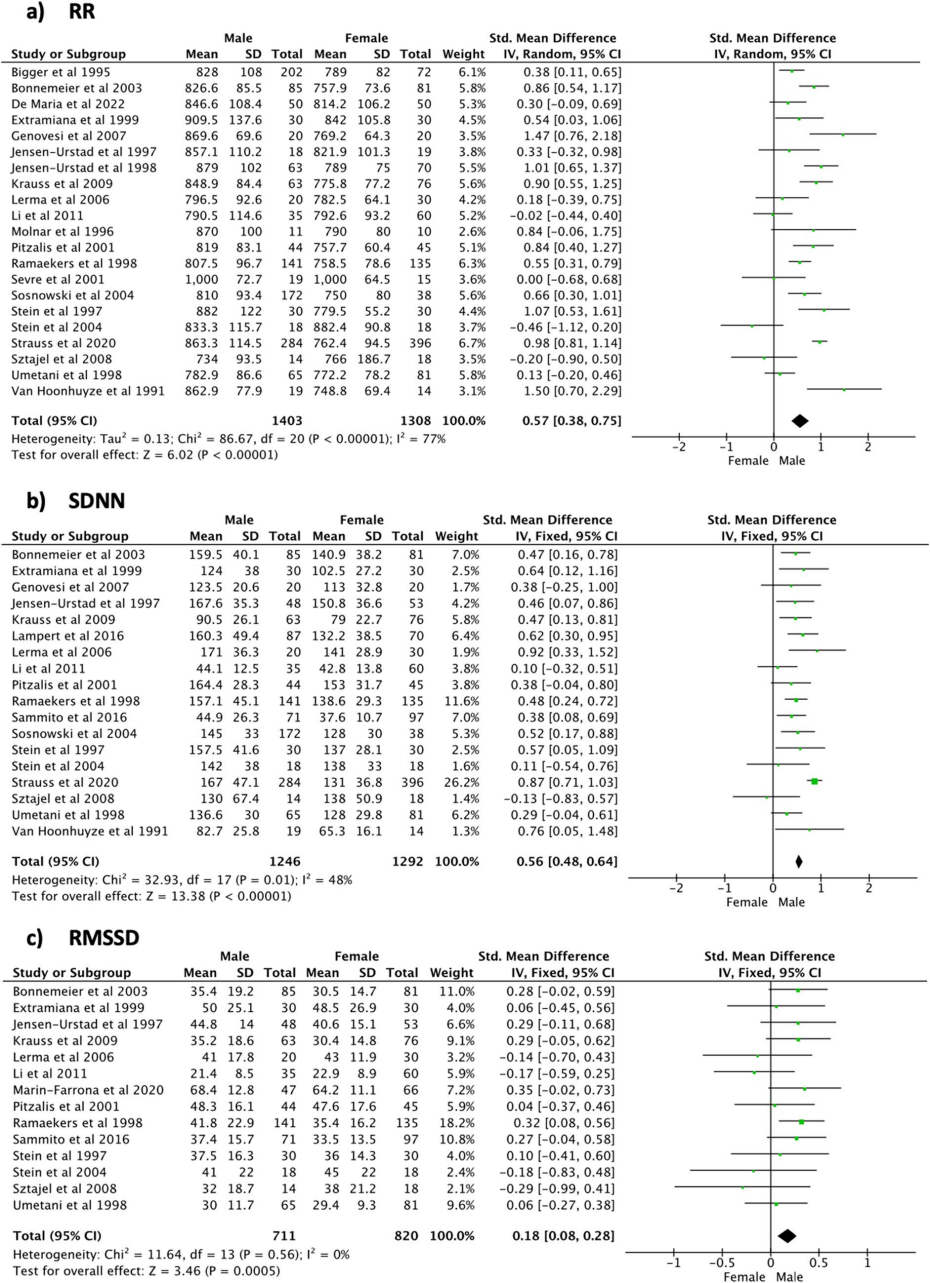
Time domain indices—24 h

As shown in Fig. 2, meta-analyses of the time domain indices revealed that the male population had a significantly longer RR interval (SMD [95% CI] 0.57 [0.38, 0.75], *p* < 0.001, 2711 subjects, 21 studies), a higher SDNN (SMD [95% CI] 0.56 [0.48,0.64], *p* < 0.001, 2538 subjects, 18 studies) and a higher RMSSD (SMD [95% CI] 0.18 [0.08, 0.28], *p* < 0.001, 1531 subjects, 14 studies) compared to the female population. All these indices showed low heterogeneity levels (*I*^2^ < 49%), except for the RR interval (*I*^2^ = 77%).

**Fig. 3:**
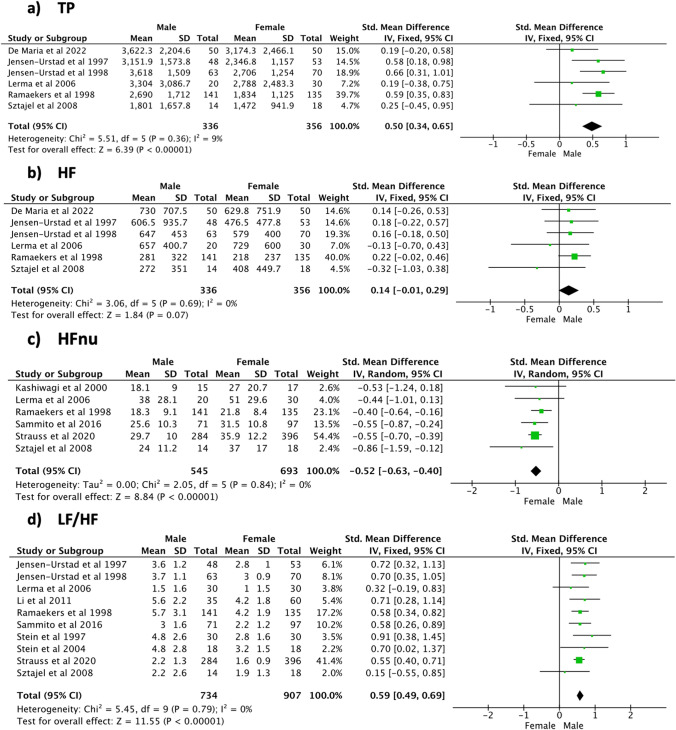
Frequency domain indices—24 h

Regarding the frequency domain analyses (Fig. 3), the male population showed significantly higher TP (SMD [95% CI] 0.50 [0.34, 0.65], *p* < 0.001, 692 subjects, 6 studies) and LF/HF (SMD [95% CI] 0.59 [0.49, 0.69], *p* < 0.001, 1641 subjects, 10 studies) compared to the female population. A positive trend in favour of the male population was noticed for the HF power, but statistical significance was not reached (SMD [95% CI] 0.14 [− 0.01, 0.29], *p* = 0.07, 692 subjects, 6 studies). Instead, the female population was characterized by a significantly higher HFnu compared to men (SMD [95% CI] − 0.52 [− 0.63, − 0.40], *p* < 0.001, 1238 subjects, 6 studies). The analysis related to the frequency domain indices demonstrated low heterogeneity levels (*I*^2^ < 10%). Meta-analysis findings during 24-h recordings are summarized in Table 3.

**Table 3:** Time and frequency domain indices—24 h

Domain	Indices	Studies	Participants	Statistical methods	*I*^2^ (%)	Effect size (M vs F)	*p* value
Time	RR	21	2711	Std. mean difference (IV, random, 95% CI)	77	0.57 [0.38, 0.75]	< 0.001
	SDNN	18	2538	Std. mean difference (IV, fixed, 95% CI)	48	0.56 [0.48, 0.64]	< 0.001
	RMSSD	14	1531	Std. mean difference (IV, fixed, 95% CI)	0	0.18 [0.08, 0.28]	< 0.001
Frequency	TP	6	692	Std. mean difference (IV, fixed, 95% CI)	9	0.50 [0.34, 0.65]	< 0.001
	HF	6	692	Std. mean difference (IV, fixed, 95% CI)	0	0.14 [− 0.01, 0.29]	0.07
	HFnu	6	1238	Std. mean difference (IV, fixed, 95% CI)	0	− 0.52 [− 0.63, − 0.40]	< 0.001
	LF/HF	10	1641	Std. mean difference (IV, fixed, 95% CI)	0	0.59 [0.49, 0.69]	< 0.001

Positive effect size indicates higher values for men, while negative effect size higher values for women

### Effect sizes during daytime recordings

**Fig. 4:**
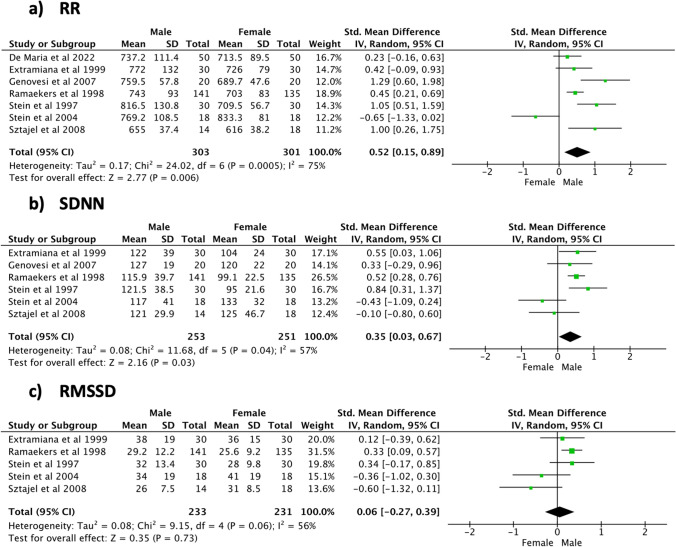
Time domain indices—daytime

During daytime (Fig. 4), significant sex differences were noticed for the RR interval (SMD [95% CI] 0.52 [0.15, 0.89], *p* = 0.006, 604 subjects, 7 studies) and the SDNN (SMD [95% CI] 0.35 [0.03, 0.67], *p* = 0.03, 504 subjects, 6 studies). No differences were found for RMSSD between female and male populations (SMD [95% CI] 0.06 [− 0.27, 0.39], *p* = 0.73, 464 subjects, 5 studies). High heterogeneity levels were observed for all the time domain indices (*I*^2^ ≥ 56%).

**Fig. 5:**
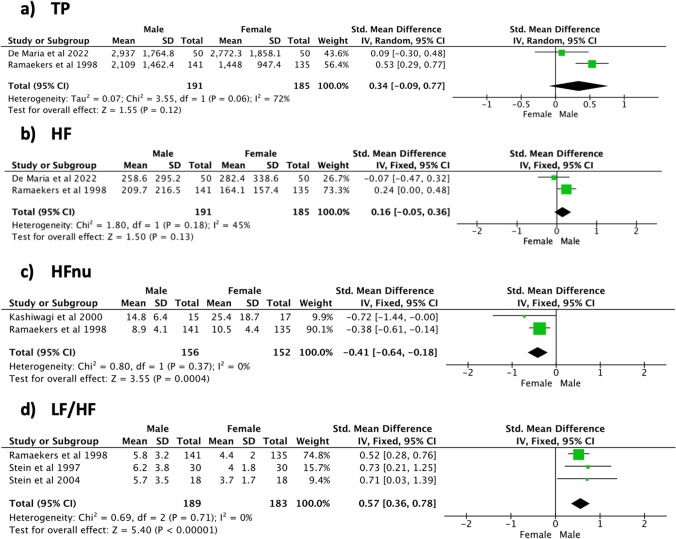
Frequency domain indices—daytime

With regard to the frequency domain outcomes (Fig. 5), the male population showed a significantly higher LF/HF (SMD [95% CI] 0.57 [0.36, 0.78], *p* < 0.001, 372 subjects, 3 studies, *I*^2^ = 0%) compared to the female one, where HFnu was significantly higher in the female population (SMD [95% CI] − 0.41 [− 0.64, − 0.18], *p* < 0.001, 308 subjects, 2 studies, *I*^2^ = 0%). No significant sex difference was observed for TP (SMD [95% CI] 0.34 [− 0.09, 0.77], *p* = 0.12, 376 subjects, 2 studies) and HF (SMD [95% CI] 0.16 [− 0.05, 0.36], *p* = 0.13, 376 subjects, 2 studies) together with relatively high heterogeneity (*I*^2^ = 45–72%).

### Effect sizes during night-time recordings

**Fig. 6:**
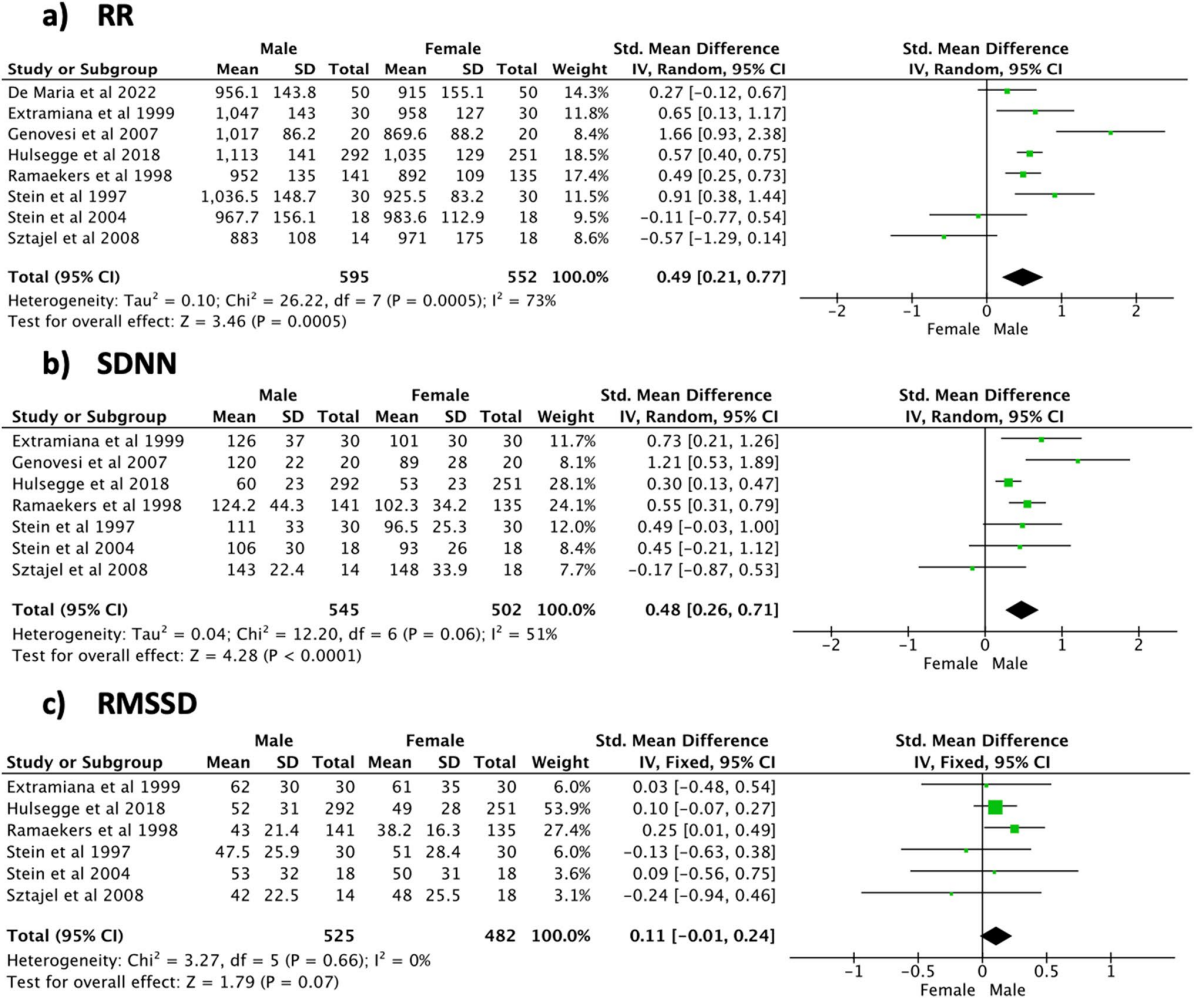
Time domain indices—night-time

**Fig. 7:**
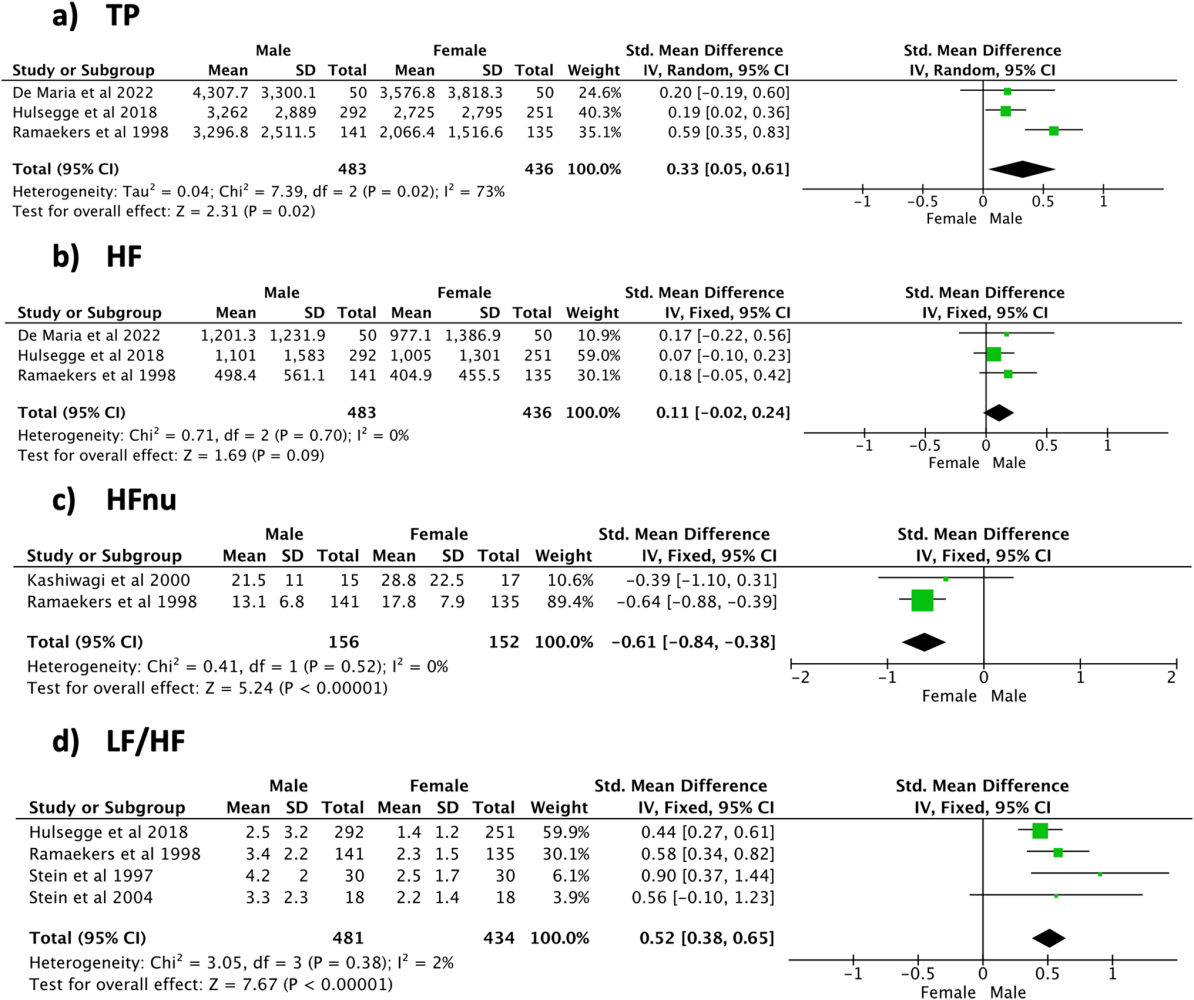
Frequency domain indices—night-time

During night-time (Fig. 6), significant sex differences and high heterogeneity were shown for the RR interval (SMD [95% CI] 0.49 [0.21, 0.77], *p* < 0.001, 1147 subjects, 8 studies, *I*^2^ = 73%) and SDNN (SMD [95% CI] 0.48 [0.26, 0.71], *p* < 0.001, 1047 subjects, 7 studies, *I*^2^ = 51%). A positive non-significant trend in favour of the male population and a low heterogeneity level was seen for RMSSD (SMD [95% CI] 0.11 [− 0.01, 0.24], *p* = 0.07, 1007 subjects, 6 studies, *I*^2^ = 0%). Examination of the frequency domain indices (Fig. 7) revealed that the male population was characterized by a significantly higher TP (SMD [95% CI] 0.33 [0.05, 0.61], *p* = 0.02, 919 subjects, 3 studies) and LF/HF (SMD [95% CI] 0.52 [0.39, 0.65], *p* < 0.001, 915 subjects, 4 studies). In addition, a significant sex difference was noticed for HFnu, with women being characterized by higher values (SMD [95% CI] − 0.61 [− 0.84, − 0.38], *p* < 0.001, 308 subjects, 2 studies). A positive, non-significant trend in favour of the male population was seen for HF (SMD [95% CI] 0.11 [− 0.02, 0.24], *p* = 0.09, 919 subjects, 3 studies). High heterogeneity levels among the included studies were shown by the meta-analyses of TP (*I*^2^ = 73%), whereas all the other frequency domain indices featured low heterogeneity (*I*^2^ ≤ 2%). Meta-analysis findings during daytime and night-time are respectively summarized in Tables 4 and 5.

**Table 4:** Time and frequency domain indices—daytime

Domain	Indices	Studies	Participants	Statistical methods	*I*^2^ (%)	Effect size (M vs. F)	*p* value
Time	RR	7	604	Std. mean difference (IV, random, 95% CI)	75	0.52 [0.15, 0.89]	0.006
	SDNN	6	504	Std. mean difference (IV, random, 95% CI)	57	0.35 [0.03, 0.67]	0.03
	RMSSD	5	464	Std. mean difference (IV, random, 95% CI)	56	0.06 [− 0.27, 0.39]	0.73
Frequency	TP	2	376	Std. mean difference (IV, random, 95% CI)	72	0.34 [− 0.09, 0.77]	0.12
	HF	2	376	Std. mean difference (IV, fixed, 95% CI)	45	0.16 [− 0.05, 0.36]	0.13
	HFnu	2	308	Std. mean difference (IV, fixed, 95% CI)	0	− 0.41 [− 0.64, − 0.18]	< 0.001
	LF/HF	3	372	Std. mean difference (IV, fixed, 95% CI)	0	0.57 [0.36, 0.78]	< 0.001

Positive effect size indicates higher values for men, while negative effect size higher values for women

**Table 5:** Time and frequency domain indices—night-time

Domain	Indices	Studies	Participants	Statistical methods	*I*^2^ (%)	Effect size (M vs. F)	*p* value
Time	RR	8	1147	Std. mean difference (IV, random, 95% CI)	73	0.49 [0.21, 0.77]	< 0.001
	SDNN	7	1047	Std. mean difference (IV, random, 95% CI)	51	0.48 [0.26, 0.71]	< 0.001
	RMSSD	6	1007	Std. mean difference (IV, fixed, 95% CI)	0	0.11 [− 0.01, 0.24]	0.07
Frequency	TP	3	919	Std. mean difference (IV, random, 95% CI)	73	0.33 [0.05, 0.61]	0.02
	HF	3	919	Std. mean difference (IV, fixed, 95% CI)	0	0.11 [− 0.02, 0.24]	0.09
	HFnu	2	308	Std. mean difference (IV, fixed, 95% CI)	0	− 0.61 [−0.84, − 0.38]	< 0.001
	LF/HF	4	915	Std. mean difference (IV, fixed, 95% CI)	2	0.52 [0.38, 0.65]	< 0.001

Positive effect size indicates higher values for men, while negative effect size higher values for women

### Meta-regression on age covariate

**Fig. 8:**
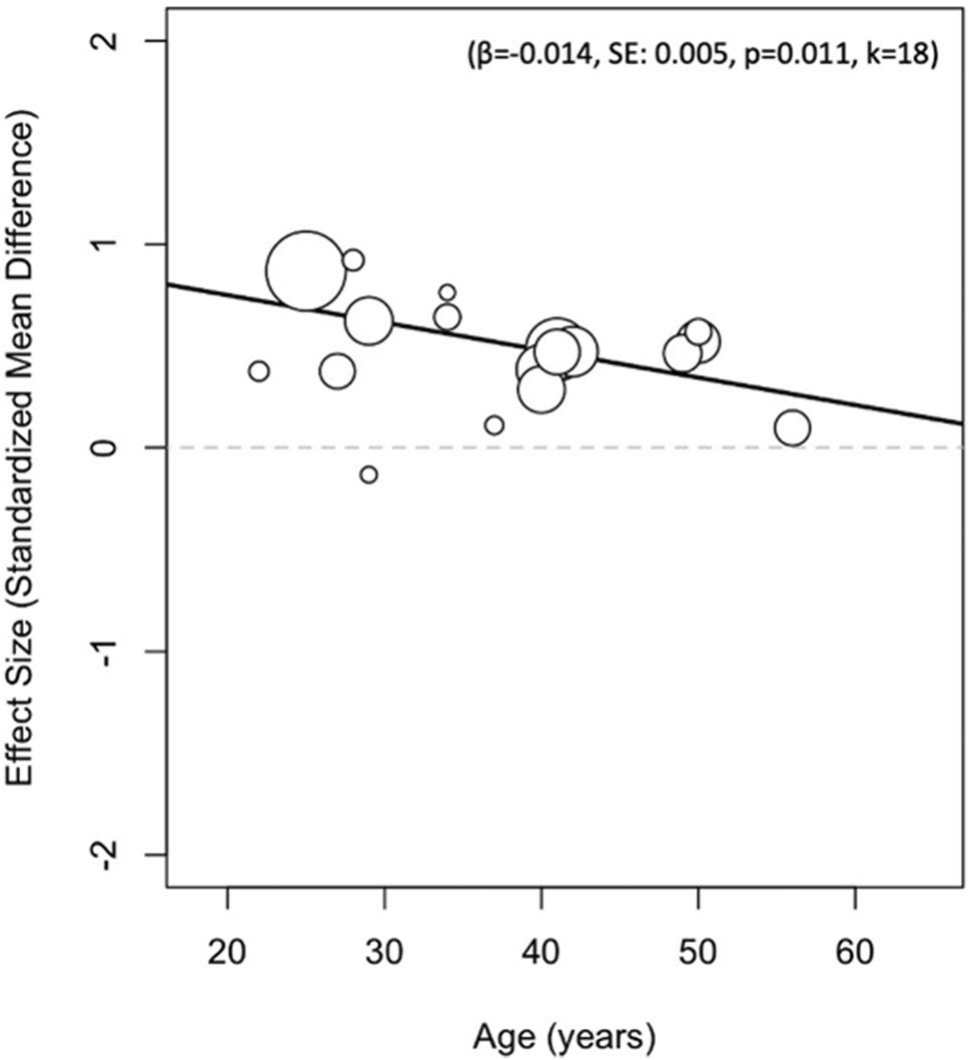
Results of meta-regression analyses on SDNN

Meta-regression was performed for RR, SDNN, RMSSD and LF/HF during 24-h recordings (see Appendix 1 in the supplementary material). These analyses revealed only a significant effect on SDNN. In detail, the previously positive effect for SDNN (i.e. higher in the male population than female population during 24-h recordings) was significantly diminished with increasing age (*β* = − 0.014, [95% CI − 0.024; − 0.003], SE 0.005, z-score − 2.56, *p* = 0.011, *k* = 18). The meta-regression results on SDNN are displayed in Fig. 8.

## Discussion

To the best of our knowledge, this was the first meta-analysis trying to separately analyse data over daytime and night-time periods in relation to sex and gender differences in HRV. Despite the limited number of studies reporting day and night information, we observed that night-time values are most consistent than daytime values in describing sex differences. As to gender, we observed that very few studies included spotted psychosocial variables and that no studies included exhaustive information about gender.

In line with a previous meta-analysis including both HRV derived from short and 24-h ECG recordings [6], the present systematic review exploring only HRV calculated over 24-h ECG recordings in healthy populations confirmed that women show a higher mean heart rate and a lower variability of the time domain indices compared to men. Our data did not definitively support an increased parasympathetic modulation in women assessed by absolute HF power, although HFnu was consistently higher in women.

### Sex-related difference in HRV

Sex-related differences in HR and HRV are a long-lasting topic in the literature. In a 1983 publication on 24-h ECG recordings from 260 healthy subjects aged 40–79, Bjerregaard found that mean HR was lower on average in male than in matched female counterparts [26]. Not only sex, but also age, smoking and leisure-time activity were found to be relevant variables in determining HR. The first studies that included the analysis of HRV date back to the early 1990s. The most accredited opinion is that of a different autonomic profile between the sexes, where women are characterized by greater vagal activity (as assessed by absolute and normalized HF powers) despite a higher resting HR and reduced variability of time domain indices [6]. However, the higher vagal modulation in women could not be the only interpretation of this phenomenon. Indeed, the lower HRV in women could also be linked to an intrinsic effect of the acetylcholine concentration on the diastolic depolarization of the pacemaker cells that generates lower HR oscillations in the presence of shorter RRs, while higher HR oscillations are produced in the presence of longer RRs [27, 28].

The results of the present review related to the most HRV indices are in line with Koenig and Thayer [6]. The result that seems to disagree with Koenig and Thayer [6] is the one related to the absolute HF power. Indeed, Koenig and Thayer [6] found a statistically significant increase in this index in women compared to men. However, the effect size was low (− 0.05 [− 0.07, − 0.03]), as it was in the present review (0.14 [− 0.01, 0.29]), suggesting that the difference between men and women is very small and possibly due to conflicting results among the included studies. It also has to be considered that in our review we focused only on HRV indices derived from 24-h ECG recordings in uncontrolled conditions, whereas Koenig and Thayer [6] included most studies concerning short ECG recordings under mixed controlled conditions, including controlled breathing and supine position. This could also explain the contrasting results with our meta-analysis, as the power in the HF band is sensitive to the respiratory rate [29]. Similar to the results of the present meta-analysis, other authors found no significant differences in absolute HF power between men and women, while a higher LF/HF ratio has been attributed to a predominant sympathetic modulation in men [30]. It remains to be established whether a small effect size of the absolute HF power is due to a similar cardiac vagal modulation in women and men or due to methodologically heterogeneous studies. Indeed, the power in the HF band could be sensitive to both the length of the period considered for analysis and the method of analysis [10]. The high heterogeneity of the methodological approaches could be responsible for the conflicting results about the comparison between men and women with regard to the power in the HF band. This bias prevents definitive conclusions from being drawn, and suggests the need for further rigorous research on this topic.

Based on classical physiology, HR in healthy individuals depends on the influence of both divisions of the autonomic nervous system on the intrinsic activity of the sinus node. Thus, HR is usually regarded as a gross marker of the autonomic interaction at the sinus node level. Accordingly, a fast HR has been associated with all-cause mortality and sudden cardiac death in the general population and in patients with heart disease [31, 32], although the strength of this association seems to be somewhat weaker in women [33].

The results of the meta-regression analysis showed that the differences between men and women are more evident in young adults than in the elderly when time domain indices, specifically SDNN, are considered. This observation suggests that the sex-related differences are attenuated by the ageing process, in agreement with previous studies [6] and possibly explained by the different hormonal settings of women in the younger age compared to menopause [18]. This is in keeping with a previous study [18] demonstrating that HRV complexity decreased more markedly in women after menopause compared to age-matched men, flattening the differences between the two sexes, probably due to the physiological reduction of oestrogens during menopause [30, 34, 35]. Intrinsic limitations of the meta-regression analysis to be disclosed are the linearity of the method and the use of the subjects’ mean age in the included studies, only a rough index of the real age, as a covariate. These limitations, together with the inability to perform the analysis according to women’s life span phase, since the majority of the included studies did not report any information, could explain non-significant findings in the other HRV indices.

### Sex hormones and HRV

Sex hormones, in particular oestrogens, may account for some of the observed phenomena in HRV. A recent review examined the link between HRV, reproductive life stages, and menopausal hormone therapy [36]. All but one study included in the review showed a decrease of the vagal dominance on the heart from the follicular to the luteal phase with higher LF power and LF/HF ratio towards the luteal phase and HF power decreasing from the follicular to the luteal phase. While a decrease in all the HRV parameters points to a reduced parasympathetic modulation, the administration of replacement therapy reverses the trend.

In addition, ovarian hormones can modulate central autonomic networks through oestrogen receptors localized in three major autonomic regions including the rostral ventrolateral medulla, the nucleus of the solitary tract, and the paraventricular nucleus of the hypothalamus [37].

Even during pregnancy, women show modifications of their autonomic profile, indicating that either hormones or hemodynamic changes or their interrelationship interfere with it [38].

All the above-mentioned evidence suggests that sex hormones have a role in determining a different autonomic control of the heart in women. Thus, a clear bias raises when interpreting HRV data in meta-analyses, since information about the hormonal stage, sex-related factors, or gender-related variables influencing the autonomic profile are not generally available.

Along this line, a recent review provided a tenable explanation for the presence of sex differences in cardiac function on a neural structural level [39]. Interestingly, this study showed a greater cortical thickness in the majority of brain regions associated with vagal outflow in women. Moreover, this study also showed that cortical thickness in brain regions associated with vagal outflow is significantly associated with HRV, but not HR [39]. Further support for the existence of core differences in the chronotropic control of the heart across sexes comes from a subsequent study from the same group showing that the association between HRV and HR is different in women and men [40]. In detail, at any given level of HRV, the heart period was found to be faster in women compared to men [40].

Today we pay the price for having long neglected the analysis of sex and gender. The lack of sex-disaggregated data may have led to a generalized misinterpretation of the data [41].

Moving to a clinical scenario, this would explain why the constant “hyperdynamic” condition of the female heart [42] does not portend a worse incidence and prognosis of cardiovascular diseases.

### Sex-related differences in HRV during night-time and daytime

Despite the paucity of studies separately assessing the differences between male and female subjects during daytime and night-time, interesting results derived from the present review. Differences between men and women, in particular concerning HRV indices in the frequency domain, were more evident during night-time compared to daytime. The main reasons should be attributed to the uniformity and standardization of the night-time period in terms of activity that characterizes all the studied subjects [43]. During the night-time period, all the subjects are supposed to sleep, the influence of the physical activity is minimized and the influence of different respiratory patterns is also attenuated. However, the observed results should also be a consequence of sex-related differences in sleep regulation. Previous studies suggested opposite results comparing women and men during wakefulness and sleep, men being characterized by greater vagal modulation during daytime compared to women, but less vagal modulation during rapid eye movement sleep [8]. Therefore, the aforementioned observations suggest that future studies should try to separately analyse daytime and night-time periods, possibly selecting the periods for analysis based on the activity that subjects are performing during the Holter ECG recording. This could be achieved using a manual or electronic diary where activities are precisely documented in order to select periods for analysis in accordance. For instance, during night-time attention should be paid to the time in bed and the number and length of the awakenings. Of course, this approach is not free from limitations, as a comprehensive sleep analysis should consider the recording of a complete polysomnography to describe sleep stages and respiratory activity [44]. However, the strategy of selecting periods for analysis based on reported activities is an attempt to standardize the analysis and limit bias with a simpler experimental setup. The same approach could be used also during the daytime to study the effect of precise activities, for instance, working activities, periods of relaxation or physical exercise.

### Gender and HRV

The results of the present review underlined that the effect of gender on HRV is very poorly investigated, in the presence of ever-increasing importance of this topic [45]. Indeed, less than 30% of the studies included in this review reported some information about gender-related factors. However, none of the studies reported exhaustive information about gender, a concept that should consider more than one factor (such as ethnicity, socio-economic factors, gender identity and geographical location). In addition, sex-disaggregated data are sometimes omitted; it is noteworthy that only 56% of the studies separately reported the age of men and women.

As to the real implementation of gender-related aspects in HRV, our results strongly encouraged the inclusion of gender-related variables when designing new experimental protocols.

### Limitations

This review with meta-analysis has some limitations that deserve mention. Although the broad search string and the literature search were performed in three pertinent electronic databases by two independent reviewers, it is not possible to exclude that some articles have been missed. Also, given the paucity of published data for daytime and night-time analyses, the meta-analyses were based on the results from a limited number of heterogeneous studies. In addition, the paucity of information about methodological details, such as sampling rate or appropriate description of the analytical methods for ECG analysis, limits possible speculation about the comparability of the included studies in terms of methodological adequacy.

## Conclusion

This updated review aimed to assess sex differences in HRV assessed over 24-h ECG recordings, with a particular focus on day and night differences and gender variables. We confirmed the sex-related differences in both the time and frequency domain of HRV, with women being more tachycardic than men and with a lower variability of the time domain indices.

In the night-time period, the aforementioned differences between women and men seem to be more pronounced, possibly due to a standardization of the state of the subjects during the night. Of interest, very few studies reported scattered data about gender factors and almost no studies reported exhaustive information about them.

Therefore, we point out the need for future research to propose a methodological approach to study gender in HRV, promoting the broad inclusion of gender-related aspects and standardized and/or precisely described procedures to collect and analyse the ECG recordings in relation to subjects’ activities.

### Supplementary Information

Below is the link to the electronic supplementary material.

Supplementary file1 (DOCX 20 KB)

## Data Availability

Not applicable.
