# Supplementary material for: Day and night heart rate variability using 24-h ECG recordings: a systematic review with meta-analysis using a gender lens
Source: Clin Auton Res. 2023 Aug 4;33(6):821–41. doi: 10.1007/s10286-023-00969-3 (PMC10751261; doi:10.1007/s10286-023-00969-3)
Supplement: Supplementary file 1 — Supplementary file1 (DOCX 20 KB) [file 10286_2023_969_MOESM1_ESM.docx]

**Appendix 1. Meta-regression results on HRV indices during 24-hour recording using age as exploratory variable**

| **Domain** | **Indices** | **β** | **SE** | **Z-score** | **p-value** | **k** |
| --- | --- | --- | --- | --- | --- | --- |
| *Time* | RR | -0.011 | 0.001 | -1.091 | 0.275 | 19 |
|  | SDNN | -0.014 | 0.005 | -2.560 | 0.011* | 18 |
|  | RMSSD | 0.003 | 0.008 | 0.340 | 0.734 | 15 |
| *Frequency* | LF/HF | 0.007 | 0.005 | 1.374 | 0.169 | 10 |
